# Supplementary material for: Risk of systemic lupus erythematosus flare after COVID-19 hospitalization: A matched cohort study
Source: PLoS One. 2024 Oct 10;19(10):e0309316. doi: 10.1371/journal.pone.0309316 (PMC11466388; doi:10.1371/journal.pone.0309316)
Supplement: S1 Fig — (PDF) [file pone.0309316.s001.pdf]

1) Temporal distribution of the COVID-19 episodes

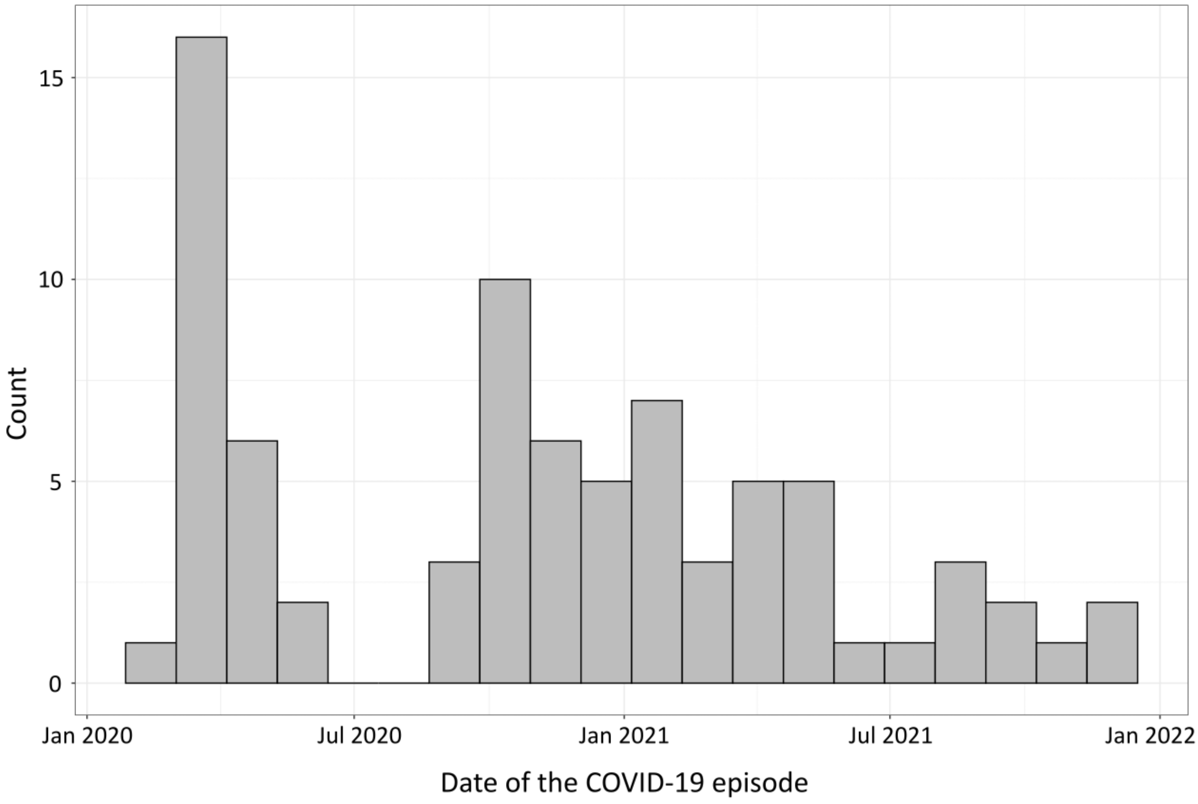

Figure S1: Temporal distribution of the COVID-19 episodes.
