# Supplementary material for: Risk of systemic lupus erythematosus flare after COVID-19 hospitalization: A matched cohort study
Source: PLoS One. 2024 Oct 10;19(10):e0309316. doi: 10.1371/journal.pone.0309316 (PMC11466388; doi:10.1371/journal.pone.0309316)
Supplement: S2 Fig — (PDF) [file pone.0309316.s002.pdf]

Post-hoc analyses included during the reviewing process.

During the reviewing process, we were asked to perform the main analysis using 3 and 12 months of follow-up. Here are the results of these analyses:

A. Using a 3-months follow-up

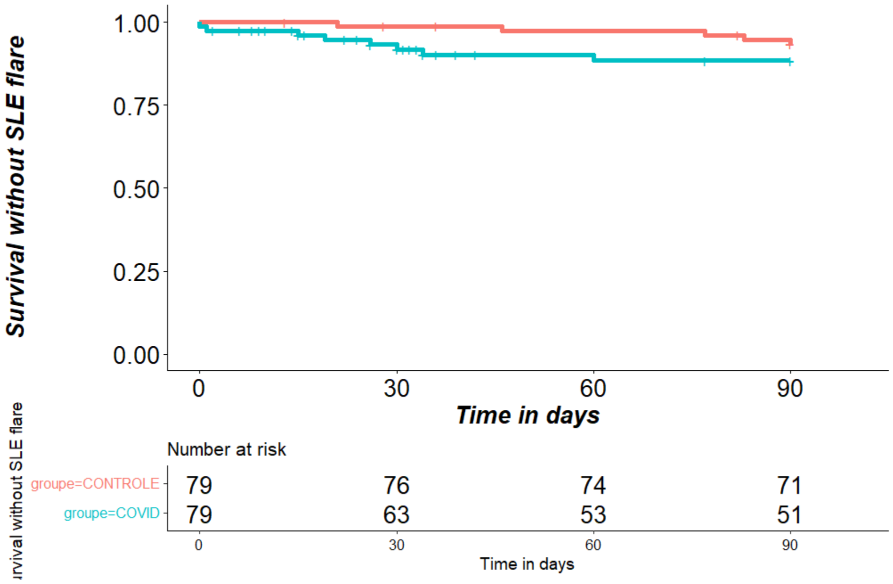

Figure S2 -a: Kaplan-Meier curve of the flare-free survival using 3 months of follow-up.

Using 3 months of follow-up, the marginal Cox model adjusted for a history of lupus nephritis and for the use of HCQ at time 0 calculated a hazard ratio of 2.45 [0.73-8.23].

B. Using a 12-months follow-up

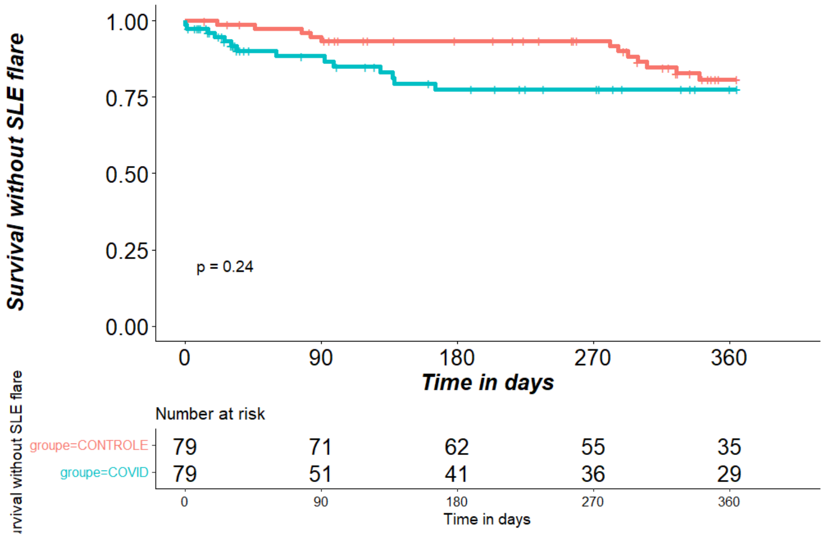

Figure S2-b: Kaplan-Meier curve of the flare-free survival using 12 months of follow-up.

Using 12 months of follow-up, the marginal Cox model adjusted for a history of lupus nephritis and for the use of HCQ at time 0 calculated a hazard ratio of 1.69 [0.84-3.38].
