# Supplementary material for: Risk of systemic lupus erythematosus flare after COVID-19 hospitalization: A matched cohort study
Source: PLoS One. 2024 Oct 10;19(10):e0309316. doi: 10.1371/journal.pone.0309316 (PMC11466388; doi:10.1371/journal.pone.0309316)
Supplement: Attachment — Submitted filename: response_to_reviewers.docx [file pone.0309316.s003.docx]

**Risk of systemic lupus erythematosus flare after COVID-19: a matched cohort study.**

**Revision #1**

**Point by point response to Reviewers’ comments.**

***Reviewer #1: The authors tried to demonstrate the increased risk of systemic lupus erythematosus flare after admission for COVID-19 in a multicentre matched cohort study.***

***1. Design: the flare rate can be influenced by many factors, especially after an infection event. If the flare risk is increased after COVID-19 infection by a short period, such as 6 months in the manuscript, the rate would be back to baseline level thereafter. It is more convincing if the authors can present these data.***

Response: Thank you for this relevant point. We agree with Reviewer 1 that if our hypothesis is correct, the flare rate should return to baseline after a certain period. We chose 6 months of follow-up *a priori* because this would let us have enough follow-up for all the included patients and because patients with SLE have usually a visit with their physician at least once every 6 months.

According to that comment, we looked at the risk of flare using 12 months of follow-up. You can see here the Kaplan-Meier curves of the flare-free survival.

We observed that, according to our hypothesis, the flare rate of the COVID-19 negative patients goes back to baseline. We added this analysis in the electronic supplementary materials of the paper.

***2. Besides from “tapering the IS”, changing prednisone could also be a common clinical practice after COVID-19 infection, how do you minimize the influence of this confounder?***

Response: As shown in the table S1 in the ESM, 23 (28.8%) of the COVID patients were treated with dexamethasone for their COVID episodes. Conversely, a decrease in the corticosteroid daily dose was considered as an “IS tapering”. As shown below, we did not find that a dexamethasone treatment or a tapering of the IS treatment was associated with the risk of flare using our competing risk analysis:

|  | Risk of SLE flare | |
| --- | --- | --- |
|  | **sdHR [95 CI]** | **p** |
| Tapering of IS drugs during COVID | 0.86 [0.23-3.18] | 0.86 |
| Dexamethasone treatment for COVID | 2.18 [0.76-6.29] | 0.15 |

This analysis has been added in table 2 in the manuscript.

***3. How do you choose matching factors? Such as chronic kidney disease, end-stage renal disease. Besides, CKD and ESRD were not independent factors.***

Response: This is of course a very important point. We wanted to use matching criteria related to the SLE “phenotype” and to the risk of flare. We chose age, sex and a marker of organ damage (CKD/ESRD) and a marker of SLE activity (dsDNA titer and C3 titer) because we believe that these variables match this definition and because they were available in our database. We agree that CKD and ESRD are not independent. We thought that renal transplantation and chronic haemodialysis really change the risk for a SLE patient to experience flare, we wanted to be sure that the number of patients with ESRD was similar within the two groups. Of note, patient with ESRD were not mixed with patients with CKD for the matching. We built a three-level variable: “No CKD/ESRD” / “CKD without ESRD” / “ESRD” and use this variable to perform the matching.

***4. When you define normal or abnormal “lupus biology”, which is not a common terminology, did you mean SACQ or SQCQ?***

Response: Thank you for raising this point. In this variable, we looked only at the biological markers. Therefore, we think that using the SACQ/SQCQ terminology would not be very relevant. However, and according to your comment, we modified the term “lupus biology” to the one of “serological activity” throughout the manuscript.

***5. Table 2: there are only 14 outcomes (flare events), however more than 10 factors were incorporated in the risk models. I am afraid that overfitting will occur****.*

Response: Indeed, we observed only 14 flare events during follow-up. However, we did not include 10 factors in the risk models. In the main model (i.e. the marginal Cox proportional hazard model), we have included only three variables: the exposure group (“COVID”/”no COVID”, the use of hydroxychloroquine at baseline and a history of lupus nephritis. And, for the competing risk models, we ran only univariate analyses.

***Reviewer #2: This is a retrospective matched cohort study investigating the relationship between COVID-19 infection and subsequent SLE flare in patients with established SLE. The authors utilized a database with clinical and administrative data that included 11 million patients seen in 39 different hospitals in Paris, France. Patients with SLE and COVID-19 infection between March 2020 and December 2021 were identified by ICD10 code and verified by chart review. The outcome of interest was physician diagnosed SLE flare (with associated medication change) within the 6 months after COVID-19 infection. Exposed patients (i.e. those who had COVID-19 infection) were matched by age, gender, renal disease and lupus biology 1:1 with unexposed SLE patients and rates of SLE flare were compared. The authors found that patients who were hospitalized due to COVID-19 infection had a higher risk of SLE flare than matched unexposed patients (HR 3.79 after adjusting for differences in HCQ use and h/o lupus nephritis).

This is a well-designed matched cohort study, with the major limitation being surveillance bias in the exposed group. It is reasonable to assume that patients who were hospitalized would be more likely to connect with their providers and have lupus flares captured in the official record. This point is mentioned as point 4 in the limitations section of the manuscript but could be further emphasized. Other points as below.***

Response: Thank you for your positive comment. We agree, as we already acknowledged that the risk of a surveillance bias is one of the main limitations of the study. According to this query, we modified the discussion section to put this limitation as the first one. However, we would like to point out that, we have all the tertiary, academic medical centres of the Paris area in our database, and that we can reasonably assume that almost all patients with SLE are followed in these centres. Then, it seems quite unlikely that we could have “missed” a flare.

***- Why was six months chosen as the follow up time frame? Please provide a rationale. If the anti-interferon biology is the proposed mechanism for SLE flare, this seems like a long time for a flare to be potentially causally associated with the infection.
- Please include a sensitivity analysis with a 3 month follow up time frame for flares as this seems biologically more plausible***

Response: Thank you for this interesting point. Reviewer #1 also raised commented on the length of the follow-up. We chose 6 months of follow-up *a priori* because this would let us have enough follow-up for all the included patients and because patients with SLE have usually a visit with their physician at least once every 6 months. However, we don’t know through which mechanisms a viral infection could trigger a flare and therefore after which delay the maximum risk of flare after COVID should be expected. According to that comment, we ran the analysis with a global censoring after 3 months. Here is the Kpaln-Meier curve:

Using this length of follow-up, we have calculated a hazard ratio of 2.45 [0.73-8.23] that failed to reach significance (p=0.15) using the same model that the one of the main analysis. We have included this analysis in the electronic supplementary materials of the manuscript.

***- Is there data available on what SLE medications hospitalized patients were discharged on? Or, data on how often immunosuppression was discontinued in these patients? This would be useful to know and could influence interpretation of the results (i.e. if patients who were hospitalized had their immunosuppressive medications discontinued it would not be surprising that they flared, and the flare would not be directly related to their COVID-19 infection).***

Response: Of course, we agree that the modification of the immunosuppressive treatment during the COVID-19 episode could be a factor explaining at least a part of the risk for a post-COVID flare. We added the number of patients with a tapering of their immunosuppressive treatment during the viral infection in the table summarizing the characteristics of the COVID episode. We have already studied this parameter in our analysis of the factor influencing the risk of flare after a COVID-19 episode in SLE patients (Table 3 of the main manuscript). The sdHR for the risk of lare associated with this variable was 0.86 [0.23-3.18] (p=0.86). Therefore, we did not observe any significant effect of a decrease in the immunosuppressive treatment during the COVID episode. However, we had only 14 flare events so it is likely that we could be underpowered in this analysis. We added a sentence in the discussion section to better underline this :” *The limited number of patients may have particularly affected the analysis of anti-dsDNA titers after the index date or the search for risk factors for flare that are both based on restricted subsets of patients that could be underpowered*.”.

***- Details of COVID-19 infection in general are useful and would recommend including them in the main manuscript rather than supplemental materials; this will help readers understand the severity of the infection (i.e. 66% required oxygen, 25% had CT-severity scores of 25-50%).***

Response: According to that comment, we added the table summarizing the characteristics of the COVID-19 infection in the main manuscript.

***- Why were two patients of the 81 unmatched? Please clarify in the manuscript.***

Response: Two COVID patients remained unmatched because they were very particular (young male with ESRD), so they did not have any similar counterpart in the non-exposed pool. We clarified that in the manuscript.

***- Why was C4 levels not included as a parameter of lupus biology?***

Response: Thank you for raising this point. We included only C3 levels and not C4 because C3 is usually considered as a more informative marker of the lupus serological activity (Semin Arthritis Rheum 2020 Oct;50(5):1081-1086.doi: 10.1016/j.semarthrit.2020.06.009.). Since complement level was included in the matching procedure, we wanted to use only one of its components to decrease the risk of missing or contradictory values.

***- Please reword the conclusion in the abstract; as written it overstates causality, which cannot be demonstrated in this study design.***

Response: According to this comment, we have rephrased the sentence in the conclusion of the abstract to: *“COVID-19 is associated with an increased risk of flare in SLE.”*

***- On page 17, please remove line 357-358. It is anecdotal that a causal link is suggested by clinicians and a bit misleading.***

Response: According to this comment, we removed the problematic sentence.

***- The discussion would benefit from further literature review and context, specifically the rates of SLE flares observed after viral infections that are not COVID19. There is only one reference on rates of flare after influenza infection. It is common wisdom that patients with SLE flare in the setting of viral infections (EBV, rhinovirus etc); how do rates of flare following COVID19 compare to rates with other viruses and why might they be different or the same?***

Response: We agree on the fact that the discussion would benefit from further literature review. We have added several new references in the discussion section, and especially studies relative to the risk of post viral flare.
