# Supplementary material for: Risk of systemic lupus erythematosus flare after COVID-19 hospitalization: A matched cohort study
Source: PLoS One. 2024 Oct 10;19(10):e0309316. doi: 10.1371/journal.pone.0309316 (PMC11466388; doi:10.1371/journal.pone.0309316)
Supplement: Attachment — Submitted filename: response_to_reviewers_R3.docx [file pone.0309316.s004.docx]

**Risk of systemic lupus erythematosus flare after COVID-19 hospitalization: a matched cohort study.**

**Revision #3**

**Point by point response to Reviewers’ comments.**

**Reviewer #4:**

***The authors have addressed the comments in the revised version, in this matched cohort study assessing risk of SLE flare after COVID.***

Response: Thank you for your comments that helped us to improve our manuscript.

**Reviewer #6:**

***The premise of study is intriguing. Most of the necessary corrections have already been made by previous reviewers.***

Response: Thank you for your positive comment.

***I have just one major comment to make. Since there was no patient communication involved in the study and everything was obtained from health records, how could the investigators ensure that the control group did not suffer from any mild covid infection not requiring hospitalization or OPD consult? Majority of COVID-19 patients improved with OTC treatment. This seems like a very significant flaw in the methodology that makes the results of the study unreliable. This should be included in the discussion.***

Response: This is indeed a very relevant point. We agree that even if we are sure to have all the COVID-19 hospitalizations, we cannot be sure that the control group did not suffer from any mild COVID-19. However, we disagree on the fact that this is a very significant flaw since we clearly defined the exposure. We considered symptomatic, hospitalized form of COVID-19 as out exposure of interest and not any COVID-19. Therefore, our conclusion is that a short-term follow-up is warranted after hospital discharge for SLE patients hospitalized for COVID-19. Our hypothesis is that it is the exaggerated immune response seen in severe COVID-19 that may be responsible for the risk of SLE flares.

According to Reviewer’s comment, we have added a sentence in the discussion section to better underline that: *“we cannot be sure that the control group did not suffer from any mild covid infection not requiring hospitalization or outpatient visit.”*

***Also, the study only involves participants who were hospitalized. The results cannot be generalized for all covid exposed SLE patients. The title of the study should be changed to ‘Risk of SLE flare after Covid-19 hospitalization’ and same correction should also be made in conclusion in abstract.***

Response: We agree with Reviewer#6 on that point. We have modified the title and the abstract of the paper consequently.
